# Intraoperative Brief Electrical Stimulation of the Spinal Accessory Nerve (BEST SPIN) for prevention of shoulder dysfunction after oncologic neck dissection: a double-blinded, randomized controlled trial

**DOI:** 10.1186/s40463-017-0244-9

**Published:** 2018-01-23

**Authors:** Brittany Barber, Hadi Seikaly, K. Ming Chan, Rhys Beaudry, Shannon Rychlik, Jaret Olson, Matthew Curran, Peter Dziegielewski, Vincent Biron, Jeffrey Harris, Margaret McNeely, Daniel O’Connell

**Affiliations:** 1 Division of Otolaryngology-Head & Neck Surgery, University of Alberta, Edmonton, Canada; 2 Department of Physical Rehabilitation Medicine, University of Alberta, Edmonton, Canada; 3 Department of Physical Therapy, University of Texas, Arlington, Texas USA; 4 Division of Plastic Surgery, University of Alberta, Edmonton, Canada; 5 Department of Otolaryngology, University of Florida, Gainesville, FL USA; 6 Faculty of Rehabilitation Medicine, University of Alberta, Edmonton, Canada

**Keywords:** Neck dissection, Electrical stimulation, Head neck cancer, Nerve regeneration, Axonal regeneration, Spinal accessory nerve

## Abstract

**Background:**

Shoulder dysfunction is common after neck dissection for head and neck cancer (HNC). Brief electrical stimulation (BES) is a novel technique that has been shown to enhance neuronal regeneration after nerve injury by modulating the brain-derived neurotrophic growth factor (BDNF) pathways. The objective of this study was to evaluate the effect of BES on postoperative shoulder function following oncologic neck dissection.

**Methods:**

Adult participants with a new diagnosis of HNC undergoing Level IIb +/− V neck dissection were recruited. Those in the treatment group received intraoperative BES applied to the spinal accessory nerve (SAN) after completion of neck dissection for 60 min of continuous 20 Hz stimulation at 3-5 V of 0.1 msec balanced biphasic pulses, while those in the control group received no stimulation (NS). The primary outcome measured was the Constant-Murley Shoulder (CMS) Score, comparing changes from baseline to 12 months post-neck dissection. Secondary outcomes included the change in the Neck Dissection Impairment Index (ΔNDII) score and the change in compound muscle action potential amplitude (ΔCMAP) over the same period.

**Results:**

Fifty-four patients were randomized to the treatment or control group with a 1:1 allocation scheme. No differences in demographics, tumor characteristics, or neck dissection types were found between groups. Significantly lower ΔCMS scores were observed in the BES group at 12 months, indicating better preservation of shoulder function (*p* = 0.007). Only four in the BES group compared to 17 patients in the NS groups saw decreases greater than the minimally important clinical difference (MICD) of the CMS (*p* = 0.023). However, NDII scores (*p* = 0.089) and CMAP amplitudes (*p* = 0.067) between the groups did not reach statistical significance at 12 months. BES participants with Level IIb + V neck dissections had significantly better ΔCMS and ΔCMAP scores at 12 months (*p* = 0.048 and *p* = 0.025, respectively).

**Conclusions:**

Application of BES to the SAN may help reduce impaired shoulder function in patients undergoing oncologic neck dissection, and may be considered a viable adjunct to functional rehabilitation therapies.

**Trial registration:**

Clinicaltrials.gov (NCT02268344, October 17, 2014).

## Background

Head and neck cancer (HNC) commonly presents in the third and fourth decade of life. Treatment choices in this cancer patient population should consider the potential for many remaining working years [1, 2]. Survivorship, quality of life (QOL), and the goal of returning to life before cancer, have become a major focus in the care of the modern HNC patient.

Advanced HNC may be treated with primary surgical resection including Level IIB with or without Level V neck dissection [3]. Retraction and manipulation of the spinal accessory nerve (SAN) is necessary to access Levels IIb and V [4]. Furthermore, the superior 5 cm of the SAN is often completely devascularized in a Level IIb dissection in order to skeletonize all lymphatic tissues off the nerve [5]. Devascularization and retraction of the SAN can result in axonal injury, which can give rise to shoulder pain and dysfunction postoperatively, even in nerve-sparing procedures [6]. Shoulder pain and dysfunction from SAN injury has pronounced and well-documented negative effects on quality of life [7]. Furthermore, as the majority of HNC patients are still of working age, the potential ramifications of shoulder dysfunction may also result in longstanding socioeconomic consequences [8].

Over the past two decades, it has been demonstrated in both animal models and clinical trials that application of intraoperative brief electrical stimulation (BES) to transected motor and sensory nerves promotes axonal outgrowth and, thereby, enhances reinnervation [9]. In studies of motor nerve regeneration, 60 min of BES applied to the nerve at 20 Hz was shown to be as effective as continuous stimulation for 2 weeks, suggesting that BES should be a clinically viable technique [9].

The aim of this study was to assess the efficacy of BES in reducing postoperative shoulder dysfunction in HNC patients undergoing oncologic Level IIb +/− Level V neck dissection. This is the first randomized controlled trial to examine the effects of an intraoperative intervention of SAN and shoulder function.

## Methods

### Study design

The BEST SPIN trial was a randomized, double-blind placebo-controlled trial at the University of Alberta, a single tertiary care cancer center in Edmonton, Canada. Patients were recruited after referral for primary surgical treatment for head and neck cancer. Institutional ethical approval was obtained from the Human Research Ethics Board (HREB) (Pro00046671) at the University of Alberta. The trial was registered on Clinicaltrials.gov (NCT02268344, October 17, 2014).

### Participants

Participants were identified for eligibility from the Northern Alberta Head and Neck Tumor Board (NAHNTB). NAHNTB is a multidisciplinary group at the University of Alberta that reviews diagnosis and treatment recommendations for all patients treated for HNC within the catchment area of central and northern Alberta, as well as Northern British Columbia and Saskatchewan. Eligible patients were those aged >18 years with a new diagnosis of HNC undergoing oncologic neck dissection including Level IIb. Patients were excluded if they had intraoperative resection of the sternocleidomastoid or trapezius muscle or SAN, previous head and neck surgery or radiation therapy to the neck, pre-existing shoulder dysfunction, an implanted electrical device (eg. pacemaker), or pre-existing neurological or neuromuscular disease. Patients were also ineligible if they required a pectoralis major, latissimus, or scapular flap for reconstruction. Recruitment was undertaken in the University of Alberta Head and Neck Clinic during preoperative surgical education sessions. Informed written consent was obtained from each participant prior to enrolment in the study.

### Randomisation and blinding

Eligible participants were block-randomised (1:1) in groups of six to receive: 1) BES, or 2) No Stimulation (NS), on the day of surgery after initiation of general anesthesia. If bilateral oncologic neck dissection including Level IIb was planned, the neck with the most extensive nodal burden was selected for randomisation. This was determined on the basis of preoperative imaging and physical exam findings prior to randomisation. Allocation concealment was by selection from shuffled sealed opaque envelopes. Patients were enrolled by the primary author (BB) according to eligibility criteria, randomisation was performed by the clinical nurse specialist (SR), and intraoperative interventions were performed by BB who was not involved in any of the outcome assessments. SR had no further involvement in the trial. Study participants and response assessors were masked to treatment allocation, and no external cutaneous markings were evident to indicate allocated treatment.

### Procedures

Specific parameters of the BES procedure are detailed in Box 1. All patients received therapeutic Level IIb neck dissection. If palpable lymphadenopathy was noted intraoperatively, a frozen pathologic section was submitted for examination. If the lymph node was found to be positive for malignancy, Level V neck dissection was performed. Medtronic NIM® 3.0 ™ 18 mm electrodes, placed intramuscularly on the motor point of the trapezius muscle, were used to monitor electromyographic changes in the muscle before and after neck dissection to determine if a significant injury resulted from the neck dissection. A significant injury was defined as a > 10% decrease in maximum CMAP amplitude (mV) from baseline readings performed upon first identification of the SAN intraoperatively.

**Fig. 1:**
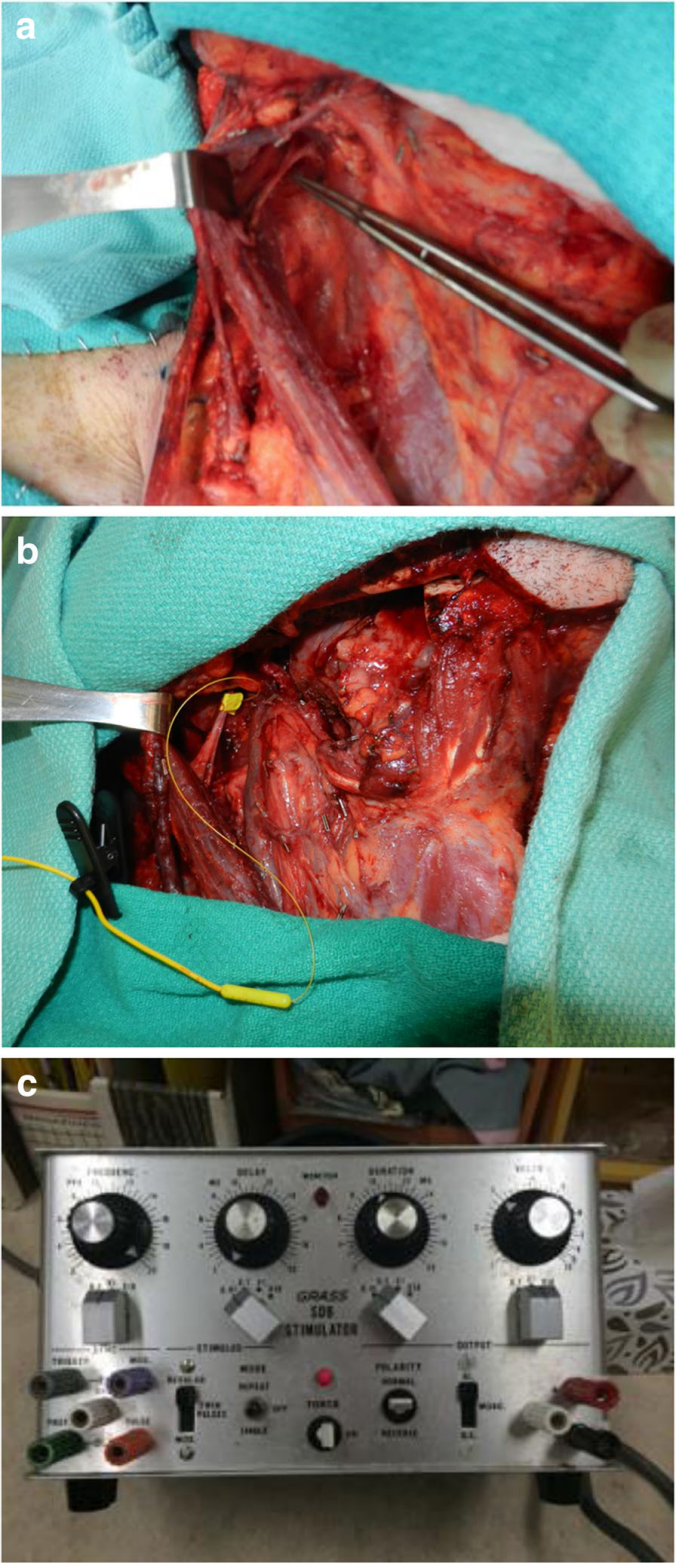
**a**-**c** BES procedure. (to be submitted as a composite figure)

Participants randomized to the NS group received no stimulation, and standard skin closure techniques were applied. For participants in the BES group, once neck dissection was complete (Fig. 1a), a 2.0 mm NIM® 3.0 automated periodic stimulation (APS) electrode cuff (Medtronic ENT, Canada) was encircled around the SAN at the proximal aspect of SAN dissection (Fig. 1b). The APS electrode was then connected to a Grass SD9 Stimulator (Grass Technologies, Quincy, MA), and the SAN was stimulated continuously at 20 Hz utilizing 0.1 msec pulses at intensities of 3–5 V for 60 min (Fig. 1c). Voltage was titrated to palpable tetanic trapezius contraction to ensure adequate stimulation. The NIM 3.0 monitoring system allowed for continued ancillary assurance of adequate stimulation. After 60 min of continuous stimulation, the APS electrode was removed and disposed of, and standard skin closure techniques were applied. During this time, other portions of the surgery were conducted. In order to ensure adequate stimulation during this time, the NIM 3.0 monitoring system was monitored, and an alert was provided if stimulation was ceased for any reason.

Prior to surgery, participants underwent baseline evaluation by a blinded physiotherapist using the Constant-Murley Score (CMS), a validated, 100-point clinical assessment scale utilizing both objective and subjective measures of shoulder function, including pain, activities of daily living (ADLs), range of motion, and strength [10, 11]. The minimally important clinical difference (MICD) for the CMS has been previously established as 10.4 points, with a standard deviation of 11 points [12–15]. This was chosen as the primary outcome due to the number of neck dissection studies previously utilizing this assessment tool, and the fact that, as the BES technique is thought to affect neuromuscular function, a functional assessment tool would be needed to evaluate this.

The participants were also evaluated with the Neck Dissection Impairment Index (NDII), a validated, 10-item, self-report questionnaire assessing neck dissection-related quality of life including evaluations of recreational, social, and self-care activities [15]. The MICD for the NDII has been previously established as 18.1 from local actuarial data [16]. Lastly, an objective evaluation of baseline SAN function was conducted using an electrophysiological measurement of maximum CMAP by an experienced, blinded neurophysiologist. Follow-up assessments of all outcomes were completed at 6 and 12 months. Twelve months was chosen as the primary endpoint follow-up measure, due to the length of the SAN, the time required for axonal regeneration along the length of this nerve, and the propensity for weakness following adjuvant treatments that may persist up to 1 year,

### Outcomes

The primary endpoint was the change in participant CMS score (ΔCMS) from baseline to 12 months after surgical treatment of the randomised shoulder. The number of participants in each group whose score decreased by greater than the MICD of the CMS was also evaluated.

Secondary endpoints included the change in participant NDII score (ΔNDII) and the change in maximum CMAP from baseline to 12 months after surgical treatment for the randomised shoulder. Adverse events (AEs) were monitored by an external Data Safety Monitoring Board (DSMB), who examined independent reports from the theatre nurse and anesthetist present on the day of the surgery. AEs were defined as any arrhythmias occurring after onset of applied BES to the SAN.

### Statistical analysis

The study was designed as a superiority study. A sample size of 21 participants in each group was sufficient to detect a difference between the BES and NS groups of 10.4 points, which represents the MICD of the primary outcome, the CMS (power of 80%; significance 5%). To compensate for a potential attrition rate of 30%, the sample size was increased to 27 per group.

Baseline demographic characteristics, tumor features, and neck dissections (Level II vs Levell IIb + V) for the two groups were compared using a Mann-Whitney U-test for continuous data and Chi-square tests for categorical data. The primary and secondary outcomes were compared between the BES and NS groups using a Mann-Whitney U-test analysis. Intention-to-treat and per-protocol analyses were undertaken. The primary outcome was also dichotomized into a ΔCMS above or below the MICD (10.4 points) at the primary endpoint of 12 months. The DSMB reviewed safety data every 6 months. Statistical analysis was performed using SPSS (Version 21.0).

The number needed to treat (NNT) was also calculated. This was performed using the number of patients whose score decreased by more than the MICD to indicate shoulder dysfunction in the NS group as the control event rate (CER) and the number of patients whose score decreased by more than the MICD in the BES group as the experimental event rate (EER).

### Role of the funding source

Funding for the study was provided by the University Hospital Foundation (UHF) Medical Research Grant Competition, a regional peer-reviewed process at the University of Alberta. The funder of the study had no role in study design, data collection, data analysis, data interpretation, or writing of the report. The corresponding author had full access to all the data in the study and had final responsibility for the decision to submit for publication.

## Results

**Fig. 2:**
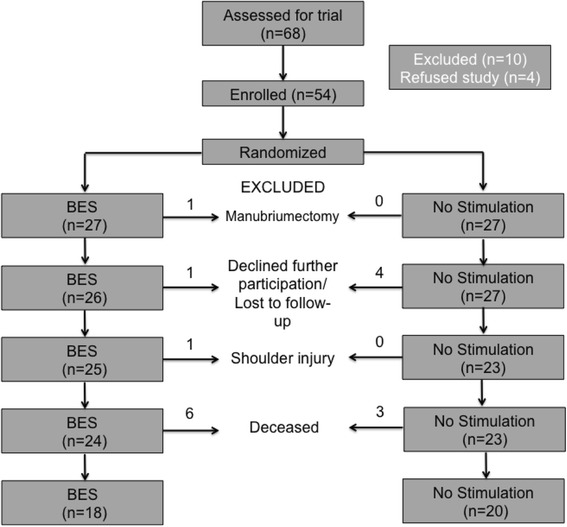
Flowchart of enrolment, intervention, allocation, and follow-up of NS and BES groups modified from the Consolidated Standards of Reporting Trials (CONSORT) 2010 Statement

Between October 6, 2014 and June 6, 2015, 68 participants were assessed for inclusion in the trial. Ten patients were not eligible due to the presence of exclusion criteria, and four patients refused participation in the trial. The remaining 54 participants were deemed eligible for the study. The median age of all participants was 60.1 years. A CONSORT diagram detailing enrolment is depicted in Fig. 2.

Mean follow-up was 257.7 days (95% CI 222.8 to 292.6, range = 0.0 to 363.0 days, σ = 131.0 days) for all patients, 254.7 (95% CI 207.2 to 302.2, range = 0.0 to 361.0 days, σ = 138.6) for the BES group and 260.5 days (95% CI 208.2 to 312.8, range 0.0 to 361.0 days, σ = 126.1) for the NS group. The most common primary tumor sites were oral cavity (33.3%) and oropharynx (24.1%). Forty-two patients (77.8%) had radiotherapy (37.0% in the BES group, 40.7% in the NS group). Fifteen patients (27.8%) had chemotherapy (18.5% in the BES group, 9.3% in the NS group) (Table 1). None of the patients required pectoralis major, latissimus flaps for wound breakdown.

**Table 1:** Demographic factors in NS and BES groups

Variable	Entire cohort	NS	BES	*P*-value
Number	54	27	27	–
Age	57.8	57.9	57.8	0.557
Gender				
Males	44 (81.5%)	21 (38.9%)	23 (42.6%)	0.555
Females	11 (20.4%)	6 (11.1%)	5 (9.3%)	
TNM stage				
Early	9 (16.7%)	5 (9.3%)	4 (7.4%)	0.352
Advanced	46 (85.2%)	22 (40.7%)	24 (44.4%)	
Tumor site				
Oral cavity	18 (33.3%)	10 (18.5%)	8 (14.8%)	0.231
Oropharynx	13 (24.1%)	7 (13.0%)	6 (11.1%)	0.532
Larynx	12 (22.2%)	7 (13.0%)	5 (9.3%)	0.323
Other	12 (22.2%)	7 (13.0%)	5 (9.3%)	0.323
Charlson Comorbidity Index	1.57	1.43	1.63	0.754
Smoking (Pack-years)	17.3	16.4	19.7	0.522
BMI (kg)	3.73	4.68	2.98	0.713
Radiotherapy	42 (77.8%)	22 (40.7%)	20 (37.0%)	0.372
Chemotherapy	15 (27.8%)	5 (9.3%)	10 (18.5%)	0.112

*NS* no stimulation, *BES* brief electrical stimulation, *BMI* body mass index

All 54 patients demonstrated a maximum CMAP decrease >10% to indicate SAN injury and eligibility for intervention. Nine and seven participants were unavailable for final analysis in the BES and NS groups, respectively, at 12 months (Fig. 2). Six-month test values were carried forward to maintain the integrity of the intention-to-treat analysis. The per-protocol analysis was undertaken using only 12-month test values. The remaining 18 and 20 participants were analyzed in the BES and NS groups, respectively. A post-hoc power calculation revealed that, with the final sample size, study power was calculated to be 99.7%.

Demographic factors, including both patient and tumor characteristics, were assessed for the entire cohort and each group separately. In 90.9% of cases, randomised patients underwent major ablative and reconstructive surgery including free tissue transfer. No differences in age, gender, TNM staging, tumor site, or Charlson Comorbidity Index (CCI) were observed (Table 1). Prognostic factors known to affect shoulder function were also evaluated between groups, and no significant difference was detected between them (Table 2). There were no significant differences between groups regarding the number of Level IIb + V neck dissections (*p* = 0.607), or the extent of surgery indicated by the number of nodes extirpated (*p* = 0.781) (Table 3).

**Table 2:** Type of neck dissection and extent of surgery in NS and BES groups

Variable	Entire cohort	NS	BES	*P*-value
N	54	27	27	–
Level IIb only	27 (50.0%)	14 (25.9%)	13 (24.1%)	0.607
Level IIb + Level V	27 (50.0%)	14 (25.9%)	13 (24.1%)	
Nodal yield	32.5	31.3	38.1	0.781

*NS* no stimulation, *BES* brief electrical stimulation

**Table 3:** ΔCMS, ΔNDII, and ΔNCS results for Level IIb + V neck dissection patients only

Variable	NS	BES	*P*-value
ΔCMS	−38.8	−6.0	0.048
ΔNDII	−35.0	−11.7	0.097
ΔNCS	−6.31	1.34	0.025

**Fig. 3:**
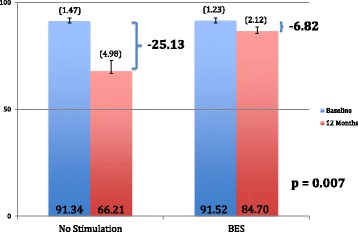
Mean ΔCMS in BES and NS groups 12 months post-neck dissection

**Fig. 4:**
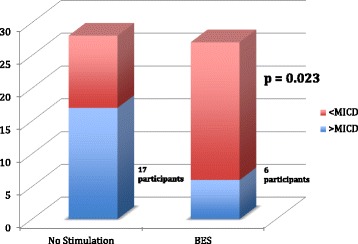
Participants with decrease in ΔCMS greater than MICD at 12 months post-neck dissection

In the intention-to-treat analysis, mean ΔCMS results 12 months post-neck dissection were −6.82 (95% CI -9.47 to −4.17, σ = 7.02, SE = 1.70) and −25.13 (95% CI -32.73 to −17.53, σ = 20.15. SE = 5.04) points for the BES and NS groups, respectively (Fig. 3). Mann-Whitney U-test demonstrated a significantly higher CMS score in the BES group at 12 months indicating significantly better preservation of shoulder function compared to the NS group (*p* = 0.007) (Fig. 3). Six (BES) and 17 (NS) patients demonstrated a decline in CMS score greater than the MICD. This difference indicates clinically relevant shoulder dysfunction in significantly fewer patients in the BES group (*p* = 0.023) (Fig. 4). The NNT for using BES for preservation of shoulder function after oncologic neck dissection was therefore calculated as 1 patient for every 2.6 patients treated with BES. In the per-protocol analysis, mean ΔCMS scores were found to be −7.25 (95% CI -9.90 to −4.60, σ = 7.03) and −24.71 (95% CI -32.14 to −17.28, σ = 19.71) for the BES and NS groups, respectively, 12 months post-neck dissection (*p* = 0.012) indicating significantly improved preservation of clinical shoulder function in the BES group (*p* = 0.012).

**Fig. 5:**
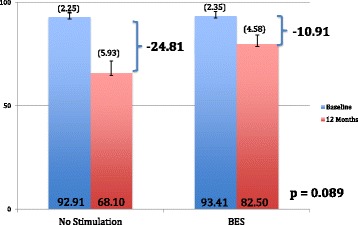
Mean ΔNDII in BES and NS groups 12 months post-neck dissection

**Fig. 6:**
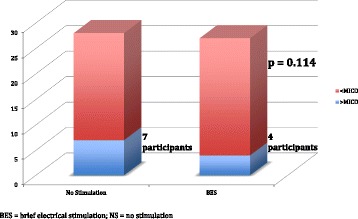
Participants with decrease in ΔNDII greater than MICD at 12 months post-neck dissection

The mean ΔNDII scores were found to be −10.91 (95% CI -16.72 to −5.10, σ = 15.40) and −24.81 (95% CI -33.50 to −16.12, σ = 23.05) points for the BES and NS groups 12 months post-neck dissection, respectively (*p* = 0.089) (Fig. 5). Four BES and seven NS patients decreased by more than the MICD of the NDII at 12 months in the BES and NS groups, respectively, indicating reduced neck dissection-related quality of life in more patients in the NS group (*p* = 0.114) (Fig. 6). When examined in the per-protocol analysis, mean ΔNDII scores were found to be −11.29 (95% CI -16.96 to −5.62, σ = 15.00) and −31.25 (95% CI -41.76 to −20.74, σ = 27.85) for the BES and NS groups 12 months post-neck dissection (*p* = 0.038) indicating better shoulder-related quality of life in the BES group.

**Fig. 7:**
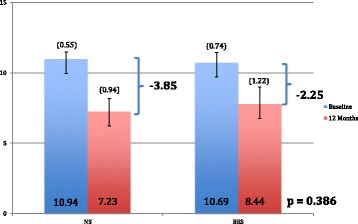
Mean ΔCMAP in BES and NS groups 12 months post-neck dissection

The mean ΔCMAPs were found to be −2.25 mA (95% CI -4.02 to −0.48, σ = 4.70) and −3.83 mA (95% CI -5.28 to −2.38, σ = 3.84) for the BES and NS groups 12 months post-neck dissection, with three and six of the six-month time-points carried forward in the BES and NS groups, respectively (*p* = 0.386) (Fig. 7). When examined in the per-protocol analysis, the mean ΔNCAs were found to be −1.13 mA (95% CI -2.78 to 0.52, σ = 4.38) and −4.52 mA (95% CI -5.79 to −3.25, σ = 3.36) for the BES and NS groups 12 months post-neck dissection (*p* = 0.067) indicating better neurophysiologic preservation of function with BES.

A subgroup analysis of ΔCMS, ΔNDII, and ΔCMAP results was undertaken in patients with Level IIb + V neck dissection (Table 3). The mean ΔCMS and ΔCMAP results were significantly higher in the BES group at 12 months (*p* = 0.048 and *p* = 0.025, respectively). No significant difference was observed in ΔNDII between groups at 12 months (*p* = 0.097).

No adverse events were observed as a result of the BES intervention. Two participants died in hospital following surgery from airway obstruction and myocardial infarction. One participant sustained a shoulder injury after an out-of-hospital fall, and one patient required a manubriumectomy for a laryngeal cancer related stomal recurrence. Six patients declined further participation or were lost to follow-up after multiple attempts to contact by the office of the surgeon and research coordinator. Nine patients were deceased at the time of final analysis.

## Discussion

The primary objective of this study was to evaluate the effect of intraoperative BES on SAN recovery following traction, compression, and devascularization injury during oncologic neck dissection. Our study demonstrated that BES was effective in reducing clinically significant shoulder dysfunction and optimizing neck dissection in patients undergoing oncologic neck dissection, specifically in those undergoing Level IIb + V neck dissection. The participants who received BES demonstrated significantly higher CMS scores post-neck dissection compared to controls. At 2.6 participants, the NNT suggests that BES is a highly efficacious treatment. Although the differences in the secondary outcomes measured did not reach statistical significance in the intent-to-treat analysis, the ΔNDII was significantly better in the BES group in the per-protocol analysis, which examined only the 12-month results for the remaining patients. As well, the ΔNDII and ΔCMAP values were congruently, if not statistically, improved in the BES group in the intent-to-treat analysis. The lack of statistical significance in the intent-to-treat analysis may be due in part to the relatively small sample size of the study, and/or to the fact that multiple 6-month values were carried forward to maintain the integrity of the intent-to-treat analysis. The study was powered based on the primary outcome measure. The significant results identified in the subgroup analysis of Level IIb + V neck dissection patients must be considered in the context of a limited sample size.

A significant body of evidence has been put forth regarding the success of BES in promoting regeneration following peripheral nerve injuries. To our knowledge, this is the first study to apply intraoperative BES to the SAN for the purposes of preventing shoulder dysfunction, specifically after oncologic neck dissection, and the first study to demonstrate a successful clinical outcome.

The application of BES to a peripheral nerve following injury was initially performed by Nix and Hopf [17] in the soleus nerve of a rabbit after a crush injury. They subsequently reported accelerated recovery of twitch force, tetanic tension, and muscle action potential in the soleus muscle. Thereafter, application of BES to the sciatic nerve proximal to a crush injury demonstrated significantly improved recovery of the toe-spread reflex [18]. Further studies demonstrated that, following application of BES to the rat femoral nerve after transection injury and primary repair, significantly increased numbers of motoneurons regenerated into the nerve branches of the rat femoral nerve when compared to a non-stimulated sham control group. This acceleration of function recovery was found to be due to accelerated sprouting of axons across the nerve repair site and not due to an accelerated rate of regeneration [19]. Subsequent studies have suggested that brain-derived neurotrophic factor (BDNF), a key molecule in activating cyclic adenomonophosphate (cAMP), and protein kinase A (PKA) which leads to downstream protein transcription necessary for neurite outgrowth, may be mediating the effect of BES in accelerating motoneuron regeneration [20]. A randomized control trial in human participants with carpal tunnel syndrome (CTS) was initiated at the University of Alberta. BES was applied for 60 min to the median nerve following carpal tunnel release and compared to a sham control group regarding both motor and sensory conduction studies. Six months after BES was applied, both the terminal motor latency and sensory nerve conduction values improved significantly faster in BES participants than in controls [21]. Thus, the findings of this study are congruent both intrinsically and extrinsically when compared to existing literature.

The NNT for BES to prevent shoulder dysfunction in 1 patient was calculated to be 2.6. For a prophylactic intervention, a widely accepted NNT is less than 40, as indicated by other well-established therapeutic interventions in medicine [22]. The shoulder morbidity incurred after Level IIB + V neck dissections, even in SAN-sparing procedures, has been well-established [23–25]. As such, controversy regarding de-escalating neck dissection techniques has ensued [26, 27]. Conversely, with the discovery of improved survival in Human Papillomavirus (HPV)-related OPC, a shift toward monotherapy in early-stage disease has been discussed, necessitating a thorough surgical approach to neck dissection in patients treated with primary surgery. Thus, the utility of BES in preventing shoulder dysfunction with a thorough dissection technique may be applicable to a larger patient population. As well, concerns often arise regarding interventions that unnecessarily prolong surgical procedures, however, the convenience of applying BES while performing other steps of major ablative and reconstructive surgeries including free tissue transfer, such as in 90.2% of analyzed cases in this study, does not alter the length of overall surgery and makes it clinically viable in the care of HNC patients.

A limitation of our study was the relatively small sample size given the potential heterogeneity in both patient characteristics and surgical procedures among patients. Examining the number of lymph nodes resected in the NS and BES groups as well as strict exclusion criteria did allow us to examine the utility of BES in the most homogenous group possible. As well, the congruency of the results across all measurements supports the significant findings in the study, despite the small sample size. However, determination of the specific effects of BES on subgroup differences, such as those undergoing chemotherapy and/or those sustaining a substantial weight loss, may require a larger sample size. Further studies are needed to target sub-groups undergoing selective or modified radical neck dissection to determine point estimates and measures of variability for the purposes of a larger Phase III study. In addition, the study was powered to detect a minimal important clinically difference (MICD) in the Constant-Murley score of 10.4 points (σ = 11) based on previous research following post-surgical rotator cuff injuries [12–15, 28, 29]. As the MICD for the Constant-Murley score following neck dissection procedures is currently not known, calculating a neck dissection-specific MICD for the Constant-Murley score is a goal of future studies. A potential confounder in the study is related to pain sustained from operative healing. However, this is unlikely to confound the outcomes, as we anticipate similar levels of pain in both groups. In addition, the intention-to-treat analysis included six-month values for all outcomes, which are likely to be adversely affected by adjuvant therapies administered within 6 months of the neck dissection. This postulation is supported by the lack of significant outcome findings at 6 months, and subsequent improved clinical shoulder function at 12 months. Lastly, this study was conducted at a single institution. A larger, multi-institutional study will need to be initiated to determine if the effect remains significant.

The clinical impact of this study is multi-dimensional. Previous human trials have demonstrated accelerated axonal regeneration histologically, as well as clinically, in motor and sensory nerves treated with BES after transection or compression injuries. The results shown in this study confirm that BES does have the ability to enhance axonal recovery in a motor nerve (SAN) post-neck dissection. As this study has demonstrated success in improving outcomes after an axonal injury with a devascularization component, BES may enhance regeneration in a more diverse population of peripheral nerve injuries than originally considered. Furthermore and most importantly, as this technique has been shown to be successful in reducing shoulder dysfunction, it may provide a useful adjunct to established functional rehabilitation approaches. As lack of treatment compliance with physiotherapy in HNC patients often negatively affects outcomes, a treatment like BES that is initiated prior to surgical recovery and delivered as a single therapeutic intervention may have considerable benefits in an oncologic patient population facing impending adjuvant therapies.

Prior to any consideration of generalized adaptation of intraoperative BES following neck dissection surgery, the reproducibility of these findings within other populations of HNC will need to be confirmed. However, as the equipment necessary to provide BES to the SAN is readily available and inexpensive in comparison to prolonged physiotherapy rehabilitation and/or missed work due to shoulder dysfunction, this can be easily facilitated. Moving forward, multi-institutional studies, cost-effectiveness, and analgesic effects [30] will need to be examined to establish the utility of this technique in preventing shoulder dysfunction in patients undergoing oncologic neck dissections.

## Conclusions

Intraoperative BES may reduce shoulder dysfunction in patients undergoing oncologic neck dissection, and can be considered an adjunct to established functional rehabilitation therapies.

## Abbreviations

ADL: Activities of daily living
AE: Adverse events
APS: Automated periodic stimulation
BDNF: Brain-derived neurotrophic factor
BES: Brief electrical stimulation
cAMP: Cyclic adenomonophosphate
CCI: Charlson Comorbidity Index
CER: Control event rate
CMAP: Compound muscle action potential
CMS: Constant-Murley Score
CTS: Carpal tunnel syndrome
DSMB: Data safety monitoring board
EER: Experimental event rate
HNC: Head and neck cancer
HPV: Human papillomavirus
HREB: Human research ethics board
MICD: Minimally important clinical difference
NAHNTB: Northern Alberta Head and Neck Tumor Board
NDII: Neck Dissection Impairment Index
NNT: Number needed to treat
NS: No stimulation
PKA: Protein kinase A
QOL: Quality of life
SAN: Spinal accessory nerve
ΔCMAP: Change in compound muscle action potential from baseline to twelve months
ΔCMS: Change in Constant-Murley Score from baseline to twelve months
ΔNDII: Change in Neck Dissection Impairment Index from baseline to twelve months
